# Independent prognostic ımpact of perineural ınvasion in breast cancer patients with residual disease after neoadjuvant chemotherapy

**DOI:** 10.1590/1806-9282.20252145

**Published:** 2026-07-10

**Authors:** Ömer Faruk Elçiçek, Özge Yalıcı, Eyyüp Çavdar, Ezel Gedik, Meltem Öznur, Okan Avcı, Erdoğan Selçuk Şeber

**Affiliations:** 1 Tekirdag Namik Kemal University, Faculty of Medicine, Department of Medical Oncology – Tekirdağ, Turkey.; 2 Tekirdag Namik Kemal University, Faculty of Medicine, Department of Medical Pathology – Tekirdağ, Turkey.

**Keywords:** Breast neoplasms, Neoplasm invasiveness, Prognosis, Neoadjuvant therapy, Survival analysis

## Abstract

**OBJECTIVE:**

Perineural invasion is a well-established adverse prognostic factor in several malignancies, yet its prognostic role in breast cancer remains controversial.

**METHODS:**

We retrospectively analyzed 400 breast cancer patients treated with neoadjuvant chemotherapy between 2012 and 2025 who had residual disease (non-pCR) at surgery. Clinicopathological variables, recurrence-free survival, and overall survival were evaluated using Cox proportional hazards models.

**RESULTS:**

Perineural invasion was identified in 42.5% of the cohort and associated with larger tumor burden, lymphovascular invasion, and nodal metastasis (p<0.01). Perineural invasion-positive status correlated with significantly inferior median recurrence-free survival (100.5 vs. 120.4 months; HR 1.77, p=0.008) and overall survival (108.5 vs. 133.6 months; HR 3.11, p<0.001). Multivariate analysis confirmed perineural invasion as a robust independent predictor of shorter recurrence-free survival and overall survival. The adverse impact was evident across all molecular subtypes, particularly HR+/HER2- and HER2+ groups.

**CONCLUSION:**

Perineural invasion is a strong independent prognostic factor for recurrence-free survival and overall survival in breast cancer patients with residual disease after neoadjuvant chemotherapy. Reporting perineural invasion status in routine pathology evaluations may aid in risk stratification for this high-risk population.

## INTRODUCTION

Breast cancer remains the leading cause of cancer incidence and mortality globally^1^. Prognosis is primarily influenced by tumor stage, grade, receptor status, and sociodemographic factors at diagnosis^2,3,4^. Perineural invasion (PNI), defined as cancer cell infiltration into the perineural space, is a known poor prognostic indicator in colorectal, prostate, and pancreatic cancers^5,6,7,8^. However, its significance in breast cancer remains controversial; while some studies report associations with poor outcomes^9,10,11^, others find no prognostic value^12^.

Given these conflicting data, the utility of PNI specifically in the high-risk neoadjuvant setting remains undefined. This study aims to evaluate the independent prognostic impact of PNI in a specific cohort of breast cancer patients with residual disease (non-pCR) following neoadjuvant chemotherapy (NAC), where pathological response strongly dictates prognosis^13^, to determine if it should be integrated into routine risk stratification.

## METHODS

### Study population

This retrospective study analyzed breast cancer cases diagnosed between January 2012 and July 2025 who received NAC and underwent surgery. The study was approved by the Institutional Ethics Committee. Staging was performed using standard imaging modalities. Clinical data were retrieved from hospital electronic records.

### Inclusion/exclusion

Included were female patients >18 years who completed NAC and surgery. Excluded were those with prior malignancies, distant metastasis at diagnosis, incomplete data, or those achieving pathological complete response (pCR). Of 559 evaluated patients, 400 met the criteria (non-pCR) and were included.

### Treatment

Patients received standard anthracycline–taxane–based NAC regimens. HER2-positive patients received trastuzumab-based therapy. Adjuvant hormonal and anti-HER2 therapies were administered according to guidelines. Surgery consisted of breast-conserving surgery or mastectomy with axillary evaluation. Adjuvant radiotherapy was administered when indicated. To ensure a homogeneous study cohort and eliminate treatment-related bias, only patients who completed the full course of standard adjuvant therapy (including 1 year of trastuzumab for HER2-positive patients) were included. Patients who discontinued therapy prematurely or developed recurrence/metastasis during the adjuvant treatment phase were excluded from the analysis.

### Pathological evaluation

pCR was defined as no residual invasive cancer in the breast and nodes. Histology was categorized as ductal or non-ductal. ER/PgR positivity was defined as >1% staining^14^. HER2 status was determined by IHC/ISH. Our hospital’s pathology laboratory accepts a Ki-67 cut-off value of 18% for luminal distinctions, and this value was used in the statistical analysis. Patients were divided into three groups based on hormone receptor and HER2 status: (1) hormone receptor-positive (ER and/or PgR positive) and HER2 negative, (2) HER2 positive (regardless of hormonal status), and (3) triple-negative (ER, PgR, and HER2 negative). PNI was examined and reported by our hospital’s pathologist. It was defined as the presence of carcinoma cells within any of the three layers of the nerve sheath in the breast parenchyma, identified through H&E (hematoxylin–eosin) and IHC analyses. The S100 marker was applied in equivocal cases. Immunohistochemical staining with S100 was performed in 37 cases (9.3%) to confirm PNI presence in morphologically equivocal slides.

### Statistical analysis

Categorical variables were presented as numbers and percentages. Univariate and multivariate analyses for factors affecting survival were performed using the Cox proportional hazards model. Recurrence-free survival (RFS) and overall survival (OS) were calculated from the date of initial diagnosis to the date of the first event, death, or the last follow-up (for cases with no observed event). Survival analyses were conducted using the Kaplan-Meier method; differences between groups were assessed using the Log-Rank test. All statistical analyses were performed using SPSS v27.0 (SPSS Inc.). The hazard ratio (HR) was reported with its corresponding 95%CI, and a p<0.05 was considered statistically significant.

## RESULTS

### Patient and tumor characteristics

The study cohort included 400 female patients with a median age of 51 years (range: 23–83). The median follow-up time was 54 months (range: 9–153 months). During this follow-up period, disease recurrence was documented in 104 (26%) patients. Among these, 22 (21.2%) were identified as locoregional recurrences, while the remainder were distant metastases. Cancer-related mortality occurred in 66 (16.5%) patients. Regarding molecular subtypes, the majority of the cohort was HR+/HER2- (257 patients, 64.3%), followed by the HER2+ group (103 patients, 25.8%) and the triple-negative group (40 patients, 10%).

PNI positivity was detected in 42.5% of patients. PNI positivity was found to be significantly higher in LVI-positive cases (80.1 vs. 16.4%, p<0.001), in patients with axillary lymph node metastasis (55.3 vs. 14.4%, p<0.001), and in cases with a tumor size greater than 2 cm (47.8 vs. 31.3%, p=0.002). Although the difference was not statistically significant (p=0.340), a notable numerical variation in PNI prevalence was observed between the HR+/HER2- (45.1%) and HER2+ (36.9%) subgroups. The detailed clinicopathological characteristics are summarized in Table 1.

**Table 1:** Clinicopathological characteristics and distribution of patients.

Clinicopathological characteristics	n (%)	PNI positive n (%)	p-value
Age			
<40 (young adult)	71 (17.8)	26 (36.6)	0.269
≥40	329 (82.2)	144 (43.8)	
Menopausal status			
Premenopausal	208 (48.0)	83 (39.9)	0.274
Peri-postmenopausal	192 (52.0)	87 (45.3)	
Histologic type			
Ductal	318 (79.5)	131 (41.2)	0.298
Others	82 (20.5)	39 (47.6)	
PgR status			
Negative	112 (28.0)	42 (37.5)	0.207
Positive	288 (72.0)	128 (44.4)	
ER status			
Negative	78 (19.5)	33 (42.3)	0.969
Positive	322 (80.5)	137 (42.5)	
Her2 status			
Negative	298 (74.5)	132 (44.3)	0.214
Positive	102 (25.5)	38 (31.4)	
Ki-67			
<18	109 (27.2)	49 (44.6)	0.543
≥18	291 (72.8)	121 (41.6)	
Histologic grade			
Grade 1–2	267 (66.8)	112 (41.9)	0.751
Grade 3	133 (33.2)	58 (43.6)	
T size			
≤2 cm	131 (32.8)	41 (31.3)	**0.002** ^s^
>2 cm	269 (67.2)	129 (47.8)	
LVI status			
Negative	238 (59.5)	39 (16.4)	**<0.001** ^s^
Positive	162 (40.5)	131 (80.1)	
Axillary status			
Negative	125 (31.2)	18 (14.4)	**<0.001** ^s^
Positive	275 (68.8)	152 (55.3)	

^s^Significant values are indicated in bold. Her2: human epidermal growth factor receptor 2; ER: estrogen receptor; PgR: progesterone receptor; LVI: Lymphovascular invasion; PNI: perineural invasion.

### Recurrence-free survival

The presence of PNI had a profound negative impact on RFS. The median RFS for the entire cohort was 112.4 months. However, when stratified by PNI status, the median RFS was significantly shorter in the PNI-positive group (100.5±5.4 months) compared to the PNI-negative group (120.4±4.4 months) (Log-Rank p<0.001). The unadjusted HR was 2.24 (95%CI 1.51–3.30).

In the univariate Cox regression analysis, PNI, along with LVI, high tumor grade, Ki67 levels, and axillary lymph node metastasis, were identified as significant predictors of shorter RFS. Conversely, ER and PgR positivity were associated with improved RFS. Upon multivariate analysis using the Enter method, PNI positivity retained its significance as a robust independent prognostic factor for RFS (HR 1.78, 95%CI 1.17–2.70, p=0.007), alongside tumor grade and axillary nodal metastasis. Notably, due to strong multicollinearity between PNI and LVI (80.1% overlap), LVI was excluded from the final multivariate models to prevent statistical artifacts and allow for the accurate assessment of PNI’s independent effect (Table 2).

**Table 2.** Univariate and multivariate analyses of factors for recurrence-free survival and overall survival.

Variable	Univariate RFS HR (95%CI)	p	Univariate OS HR (95%CI)	p	Multivariate RFS HR (95%CI)	p	Multivariate OS HR (95%CI)	p
Age (<40/≥40)	0.80 (0.50–1.29)	0.360	0.90 (0.50–1.65)	0.732	–	–	–	–
Menopause status (pre/peri–post)	1.26 (0.85–1.86)	0.247	1.00 (0.62–1.62)	0.997	–	–	–	–
Histologic type (ductal/others)	0.93 (0.58–1.50)	0.761	1.27 (0.73–2.21)	0.394	–	–	–	–
PgR status (negative/positive)	0.62 (0.42–0.93)	**0.019**	0.53 (0.33–0.87)	**0.011**	0.65 (0.32–1.32)	0.235	0.87 (0.31–2.45)	0.793
ER status (negative/positive)	0.55 (0.39–0.84)	**0.005**	0.39 (0.23–0.64)	**<0.001**	0.88 (0.41–1.88)	0.742	0.49 (0.29–0.84)	**0.009**
Ki67 (<18/≥18)	1.65 (1.03–2.65)	**0.039**	2.45 (1.27–4.73)	**0.007**	1.36 (0.83–2.23)	0.224	1.91 (0.97–3.78)	0.062
HER2 status (negative/positive)	1.10 (0.71–1.70)	0.667	1.13 (0.66–1.95)	0.655	–	–	–	–
Grade (≤2/3)	2.13 (1.44–3.14)	**<0.001**	2.96 (1.80–4.85)	**<0.001**	1.89 (1.24–2.88)	**0.003**	2.25 (1.32–3.82)	**0.003**
cT size (≤2 cm/>2 cm)	1.23 (0.81–1.87)	0.338	1.53 (0.88–2.65)	0.134	–	–	–	–
LVI (negative/positive)	2.14 (1.45–3.16)	**<0.001**	1.82 (1.12–2.97)	**0.016**	Excluded^*^	–	Excluded^*^	–
PNI (negative/positive)	2.24 (1.51–3.30)	**<0.001**	3.05 (1.83–5.07)	**<0.001**	1.78 (1.17–2.70)	**0.007**	2.60 (1.49–4.54)	**<0.001**
Axillary status (negative/positive)	2.36 (1.45–3.85)	**<0.001**	2.16 (1.20–3.91)	**0.011**	2.33 (1.37–3.98)	**0.002**	1.87 (0.97–3.62)	0.062

Significant p-values are indicated in bold. ER: estrogen receptor; PgR: progesterone receptor; HER2: human epidermal growth factor receptor 2; LVI: lymphovascular invasion; PNI: perineural invasion; RFS: recurrence-free survival; HR: hazard ratio; CI: confidence interval; OS: overall survival.

^*^LVI was intentionally excluded from the final multivariate models due to strong multicollinearity with PNI (80.1% overlap), which inflated standard errors and artificially masked the independent prognostic value of PNI.

### Overall survival

**Figure 1:**
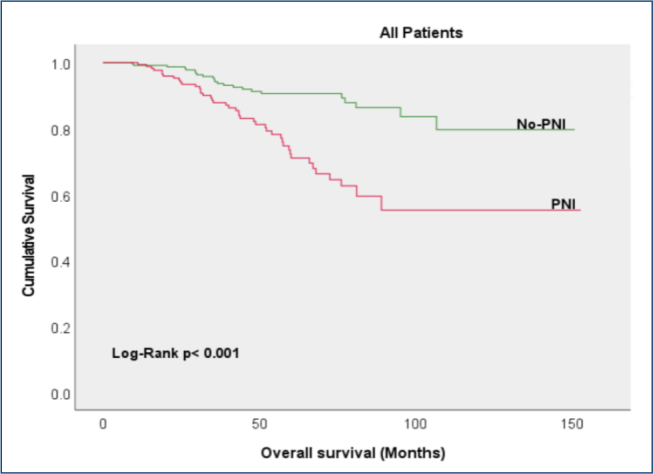
Kaplan-Meier survival curves showing the association between perineural invasion and overall survival.

Consistent with RFS findings, OS was significantly compromised in patients with PNI. The median OS was 108.5±5.8 months in the PNI-positive group, compared to 133.6±3.6 months in the PNI-negative group (Log-Rank p<0.001) (Figure 1).

Univariate analysis demonstrated that PNI positivity was associated with a more than three-fold increase in the risk of mortality (HR 3.11, 95%CI 1.87–5.19, p<0.001). Other significant factors included ER/PgR status, tumor grade, and nodal involvement. In the full multivariate Cox regression model, PNI confirmed its role as a robust independent prognostic factor for OS (HR 2.60, 95%CI 1.49–4.54, p<0.001), together with tumor grade. Interestingly, when evaluated in this comprehensive model, axillary lymph node metastasis was attenuated and lost its independent predictive value for OS (HR 1.87, 95%CI 0.97–3.61, p=0.062), as did Ki-67 (Table 2).

### Survival relationship of perineural invasion according to breast cancer subtypes

We further investigated the impact of PNI across different breast cancer subtypes. PNI prevalence was statistically similar across groups: 45.1% in HR+/HER2-, 36.9% in HER2+, and 40% in triple-negative cases (p=0.340). Survival analysis revealed that PNI positivity was associated with significantly worse RFS and OS in both the HR+/HER2- (p=0.013 for RFS, p=0.001 for OS) and HER2+ (p<0.001 for RFS, p=0.002 for OS) subgroups. In the triple-negative subgroup, while a trend toward worse survival was observed in PNI-positive patients, it reached statistical significance only for OS (p=0.046), likely due to the smaller sample size in this subgroup.

## DISCUSSION

This study provides compelling evidence that PNI serves as a robust, independent adverse prognostic factor for both RFS and OS in breast cancer. Uniquely, this analysis distinguishes itself by focusing exclusively on a high-risk cohort: patients with residual disease following neoadjuvant chemotherapy. Our multivariate analysis revealed that the presence of PNI is associated with an approximately two-fold increased risk of recurrence and a three-fold increased risk of mortality. Importantly, this prognostic value persisted independently of established risk factors such as axillary lymph node metastasis, tumor grade, and hormone receptor status. The finding that PNI remained significant while LVI did not in the multivariate model suggests that PNI may represent a distinct biological mechanism of tumor spread, potentially serving as a low-resistance route for cancer cell dissemination beyond the lymphatic system.

In the specific context of breast cancer, however, the prognostic value of PNI has historically been controversial, and the available findings are contradictory^15,16^. Early studies have reported that PNI lacks prognostic value; for example, Duraker et al. and Karak et al. reported low PNI prevalence (1.14–25.7%) and failed to demonstrate an independent prognostic role^12,17^. Similarly, an exploratory study presented at AACR 2022 reported no significance for survival^18^. These discrepancies may be attributed to methodological heterogeneity, such as evaluating unselected patient populations and relying solely on H&E staining. Conversely, recent literature supports its adverse effect. Narayan et al. reported that PNI significantly increased the locoregional recurrence risk^9^, while Hosoya et al. and Cavdar et al. demonstrated that PNI was an independent poor prognostic factor for survival^10,11^.

The frequency of PNI in breast cancer is reported to vary in the literature, typically ranging from 14 to 25.7% in unselected cohorts^9,10,11,12^. The prevalence in our study (42.5%) was notably higher. This is likely attributable to the specific selection of a high-risk population with residual disease after neoadjuvant chemotherapy, representing a biologically more aggressive tumor subset. Furthermore, a study involving oral cavity carcinomas found that the PNI-positive rate increased from 30 to 82% upon re-evaluation with immunohistochemical staining^19^. In our study, the supplementary use of S-100 staining in morphologically equivocal cases may have minimized the underdetection commonly associated with standard H&E evaluation, contributing to the observed prevalence.

The strong prognostic signal observed is biologically plausible. The persistence of PNI in post-treatment specimens implies a chemo-resistant phenotype, where perineural spaces may act as “sanctuary sites” protecting tumor cells from systemic therapy and apoptosis^20^. Thus, post-treatment PNI represents a more aggressive biological marker than in treatment-naïve settings. Furthermore, consistent with the literature, while ER and PgR positivity emerged as protective factors, the presence of PNI significantly counterbalanced this favorable effect. From a clinical perspective, these findings suggest that the presence of PNI should be routinely reported in pathology reports and considered in risk stratification. Particularly in the determination of adjuvant treatment strategies, PNI positivity can be considered a high-risk feature.

### Limitations

Our study is limited by its retrospective design and single-center nature. Furthermore, excluding patients who discontinued adjuvant therapy or experienced early recurrence introduces a potential survivorship bias. While this strict selection was essential to maintain a homogeneous cohort and eliminate treatment-related confounders, it implies that the true adverse prognostic effect of PNI might be even more pronounced than observed. Additionally, our reliance on an institutional Ki-67 cut-off of 18% for luminal distinctions lacks universal standardization, which may limit the broader generalizability of our findings. Finally, although we utilized S-100 immunohistochemistry to successfully confirm PNI in morphologically equivocal cases, relying primarily on standard H&E staining for the majority of the cohort may still underestimate the true prevalence of PNI due to potential false negatives, thereby possibly influencing our hazard estimates. Nevertheless, a significant strength of our study is the comprehensive, simultaneous analysis of numerous clinicopathological variables in a well-defined, large cohort of high-risk patients with residual disease after NAC, highlighting PNI’s critical role in identifying patients at the highest risk of progression.

## CONCLUSION

Our findings demonstrate that PNI is a significant independent prognostic factor for recurrence-free and OS in breast cancer patients with residual disease after neoadjuvant chemotherapy. Given its strong association with adverse outcomes, incorporating PNI assessment into routine pathological reporting may aid in clinical risk stratification. While it serves as a robust prognostic marker, its definitive utility in guiding adjuvant treatment strategies warrants further validation through prospective clinical trials in this high-risk population.

## Data Availability

The datasets generated and/or analyzed during the current study are available from the corresponding author upon reasonable request.

## References

[1] Bray F, Laversanne M, Sung H, Ferlay J, Siegel RL, Soerjomataram I (2024). Global cancer statistics 2022: GLOBOCAN estimates of incidence and mortality worldwide for 36 cancers in 185 countries.. CA Cancer J Clin.

[2] Waks AG, Winer EP (2019). Breast cancer treatment: a review.. JAMA.

[3] Karaboyun K, Öznur M, Yolcu A, İriağaç Y, Seber S (2023). Investigation of the effect of low-positive HER-2 on neoadjuvant chemotherapy response in hormone-positive breast cancer patients.. J Acad Res Med.

[4] Rivas FWS, Gonçalves R, Mota BS, Sorpreso ICE, Toporcov TN (2024). Comprehensive diagnosis of advanced-stage breast cancer: exploring detection methods, molecular subtypes, and demographic influences - A cross-sectional study.. Clinics (Sao Paulo).

[5] Ozaki H, Hiraoka T, Mizumoto R, Matsuno S, Matsumoto Y, Nakayama T (1999). The prognostic significance of lymph node metastasis and intrapancreatic perineural invasion in pancreatic cancer after curative resection.. Surg Today.

[6] Law WL, Chu KW (2004). Anterior resection for rectal cancer with mesorectal excision: a prospective evaluation of 622 patients.. Ann Surg.

[7] Niu Y, Förster S, Muders M (2022). The role of perineural invasion in prostate cancer and its prognostic significance.. Cancers (Basel).

[8] Shaikh M, Shirodkar S, Doshi G (2025). Unraveling the role of perineural invasion in cancer progression across multiple tumor types.. Med Oncol.

[9] Narayan P, Flynn J, Zhang Z, Gillespie EF, Mueller B, Xu AJ (2021). Perineural invasion as a risk factor for locoregional recurrence of invasive breast cancer.. Sci Rep.

[10] Hosoya K, Wakahara M, Ikeda K, Umekita Y (2023). Perineural invasion predicts unfavorable prognosis in patients with invasive breast cancer.. Cancer Diagn Progn.

[11] Cavdar E, Iriagac Y, Karaboyun K, Avci O, Oznur M, Seber S (2022). Prognostic role of lymphovascular invasion and perineural invasion in breast cancer treated with neoadjuvant chemotherapy.. Int J Hematol Oncol.

[12] Duraker N, Caynak ZC, Türköz K (2006). Perineural invasion has no prognostic value in patients with invasive breast carcinoma.. Breast.

[13] Cristófalo MM, Maesaka JY, Pereira DA, Nóbrega GB, Reis YN, Júnior JMS (2026). Neoadjuvant chemotherapy in triple negative breast cancer: a systematic review of breast and node pathologic response.. J Surg Oncol.

[14] Allison KH, Hammond MEH, Dowsett M, McKernin SE, Carey LA, Fitzgibbons PL (2020). Estrogen and progesterone receptor testing in breast cancer: ASCO/CAP Guideline update.. J Clin Oncol.

[15] Bahmad HF, Wegner C, Nuraj J, Avellan R, Gonzalez J, Mendez T (2025). Perineural invasion in breast cancer: a comprehensive review.. Cancers (Basel).

[16] McCready DR, Chapman JA, Hanna WM, Kahn HJ, Yap K, Fish EB (2000). Factors associated with local breast cancer recurrence after lumpectomy alone: postmenopausal patients.. Ann Surg Oncol.

[17] Karak SG, Quatrano N, Buckley J, Ricci A (2010). Prevalence and significance of perineural invasion in invasive breast carcinoma. Conn Med.

[18] Cox SE, Bassett RL, Yi M, Sahin A, Teshome M, Hunt K (2022). Abstract P4-07-13: an exploratory case-control study of perineural invasion in breast cancer.. Cancer Res.

[19] Kurtz KA, Hoffman HT, Zimmerman MB, Robinson RA (2005). Perineural and vascular invasion in oral cavity squamous carcinoma: increased incidence on re-review of slides and by using immunohistochemical enhancement.. Arch Pathol Lab Med.

[20] Alves MS, Damous LL, Ferreira IDS, Ferreira CDS, Sorpreso ICE, Cipolla-Neto J (2026). Melatonin’s oncostatic effects in experimental breast cancer: a systematic review of doseresponse and apoptotic mechanisms in animal models.. Clinics (Sao Paulo).

